# Exploitation of enrofloxacin-loaded docosanoic acid solid lipid nanoparticle suspension as oral and intramuscular sustained release formulations for pig

**DOI:** 10.1080/10717544.2019.1580798

**Published:** 2019-03-16

**Authors:** Yanfei Tao, Fei Yang, Kuiyu Meng, Dongmei Chen, Yujuan Yang, Kaixiang Zhou, Wanhe Luo, Wei Qu, Yuanhu Pan, Zonghui Yuan, Shuyu Xie

**Affiliations:** a MAO Key Laboratory for Detection of Veterinary Drug Residues, Huazhong Agricultural University, Wuhan, China;; b National Reference Laboratory of Veterinary Drug Residues (HZAU), Huazhong Agricultural University, Wuhan, China

**Keywords:** Enrofloxacin, docosanoic acid-solid lipid nanoparticles, suspension, oral formulation, intramuscular formulation

## Abstract

In our previous study, enrofloxacin-loaded docosanoic acid solid lipid nanoparticles (SLNs) could be effectively delivered to cells *in vitro.* In this study, its properties and exploitation as possible oral and intramuscular sustained release formulations for pigs were studied after being made into suspension. The re-dispersed time and sedimentation rate of the nanosuspension were 55 s and 1, respectively. It showed good stability when stored away from light and sustained release in pH = 7.4 PBS buffer. The suspension exhibited no irritation at the injection site and good palatability. Compared with commercial injection and soluble powder, the nanosuspension increased the bioavailability of enrofloxacin by 1.63 and 2.38 folds, and extended the mean residence time (MRT) of the drug from 11.27 and 12.33 to 37.76 and 35.15 h after intragastric and intramuscular administration, respectively. These results suggest that docosanoic acid SLN suspension (DAS) might be a promising oral and intramuscular sustained release formulation to enhance the pharmacological activity of enrofloxacin.

## 1. Introduction

Enrofloxacin is widely used to treat bacterial infections in livestock and poultry due to its strong and broad-spectrum antibacterial activity (Elsheikh et al., 2002; Elmas et al., 2007; Harada et al., 2014; Ruennarong et al., 2016). However, its poor aqueous solubility causes variable bioavailability and results in difficulties, in the design of pharmaceutical formulations (Martinez et al., 2006). Its bitter taste is unacceptable for animals, especially for pigs. The commercially available oral formulations cannot effectively mask the bitter taste and limit their clinical applications for pigs. In addition, the drug has short elimination half-life (*T*_1/2ke_) and mean residence time (MRT) in most mammals (Haritova et al., 2003). The repeated and high doses should be needed in the clinic and might result in animal inconvenience, drug resistance, and environmental contamination. Alternative formulations should be developed to mask the bitter taste, enhance the absorption and reduce the frequency of drug administration (Yang et al., 2015).

Solid lipid nanoparticles (SLNs) with excellent biocompatibility, biodegradability and stability are effective carriers for masking bitter taste, enhancing the bioavailability, and offering sustained release (Ansari et al., 2016; Luo et al., 2006; Solimana, 2017; Ji et al., 2016; Xie et al., 2008). They can be produced on a large scale and can be delivered by almost all routes, such as oral, intramuscular, subcutaneous, pulmonary, and intravenous administrations (Xie et al., 2010). Our previous study showed that tetradecanoic acid, palmitic acid, and stearic acid formulated SLNs are promising sustained release systems for enrofloxacin with enhanced bioavailability by intramuscular administration (Xie et al., 2011). In order to finally implement the clinical application, the newly prepared enrofloxacin-loaded docosanoic acid SLN with higher drug loading compared to the above three fatty acids formulated SLNs were developed (Xie et al., 2011, 2017). The docosanoic acid SLNs could more effectively delivery enrofloxacin into cells and exhibit stronger intracellular retention and enhanced activity of enrofloxacin against intracellular bacteria (Xie et al., 2017).

In this work, the enrofloxacin-loaded docosanoic acid SLN was prepared into suspension formulation (DAS). The properties, palatability and sustained release performance of the nanosuspension in pigs were investigated to explore the clinic application as an oral taste-masking and injection sustained formulation.

## 2. Materials and methods

### 2.1. Materials

Enrofloxacin of reference standard was purchased from the China Institute of Veterinary Drug Control (Beijing, China). Native enrofloxacin was obtained from Wuhan Konglong Century Technology Development Co., Ltd. (Wuhan, China). Enrofloxacin soluble powder was purchased from Dahua Nong Animal Husbandry Technology Co., Ltd (Guangzhou, China). Baytril injection solution (5%, 50 mg of enrofloxacin per ml) was purchased from Bayer (Germany). Docosanoic acid was bought from Shanghai Aladdin Biochemical Polytron Technologies Inc. (Shanghai, China). Polyvinyl Alcohol (PVA, Wt 30000-70000) was purchased from Sigma-Aldrich (St. Louis, MO, USA). Methyl alcohol and acetonitrile with high-performance liquid chromatography (HPLC) grade were purchased from Tedia (Ohio, USA). The water for HPLC was prepared with a Milli-Q system (Millipore, Bedford, MA). Other chemicals and reagents not specified in the text were of analytical grade or equivalent.

### 2.2. Animals

Thirty-three clinically healthy pigs (weighing 15–20 kg) were purchased from the Livestock and Poultry Breeding Center of Hubei Province (Wuhan, China). The pigs were kept at laboratory animal rooms at the National Reference Laboratory of Veterinary Drug Residues of Huazhong Agricultural University and provided ad libitum access to water and antibiotic-free feed for 1 week to acclimatize. The temperature and relative humidity of the housing environment were kept at 18–25 °C and 45–65%, respectively. All the animal experimental protocols in this study were approved by the Institutional Animal Care and Use Committee at Huazhong Agricultural University (approval number HZAUSW-2016-006).

### 2.3. Preparation of DAS

The nanosuspension was prepared by hot homogenization and ultrasonication method as described previously (Xie et al., 2017). Briefly, a 50 mL tube containing 0.4 g enrofloxacin and 1.6 g docosanoic acid were put in a boiling water bath. After dissolving of enrofloxacin in the melted docosanoic acid, 10 mL of 1% PVA solution preheated to 100 °C were poured into the lipid phase under magnetic stirring at 150 rpm for 3 min. The mixture was sonicated for 8 min using 3 mm microprobes with 78 watts (VCX 130Vibra-CellTM with 60% amplitude, Sonics & Materials, Inc., Newtown, CT, USA) to obtain a nanoemulsion. The hot nanoemulsion was cooled down by putting into low-temperature environment to form stable nanoparticle suspension.

### 2.4. Scanning electron microscopy (SEM)

The morphology of nanoparticles was measured using an SEM (JSM-6360LV, Japan). The nanoparticle suspension placed on the glass surface was air dried at room temperature for 5 min and then followed by oven drying. The samples after oven-drying were fixed on an SEM stub and coated with gold at 20 mA for 2 min using an auto fine coater (Ion sputter JFC 1600). After coating, the samples were observed by the SEM with a secondary electron detector with an accelerating voltage of 15 kV.

### 2.5. Determination of loading capacity (LC) and encapsulation efficiency (EE)

In order to determine the EE and LC, the nanosuspension was collected by centrifugation at 14,000 rpm (Hitachi Centrifugation CR21GIII; Hitachi Koki Co., Ltd., Japan) for 60 min at 4 °C. The free enrofloxacin in the supernatant after centrifugation was measured by Waters 2695 series High-performance liquid chromatography (HPLC) equipped with Waters 2587 UV detector (Waters Corp., Milford, MA) to determine the EE. The precipitated SLN were re-suspended in distilled water and lyophilized for 48 h (Freeze Dry System; Labconco, America) for determination of LC. The 10 mg freeze-dried nanoparticles were added in a 15 mL tube containing 10 mL acetonitrile/water solution (v/v; 1:1) and put in a boiling water bath to destroy the nanoparticles. The SLNs after heating was added to the volume of 10 mL and centrifuged at 8000 rpm for 10 min. The supernatant after filtration was injected into HPLC for analysis. The assay was repeated in triplicate by using tree batch different samples. The EE and LC were defined as follows:

$$\text{EE}\left(\%\right)=\frac{\begin{array}{c} (\text{Weight}\;\text{of}\;\text{enrofloxacin added}\\ -\text{weight of}\;\text{enrofloxacin}\;\text{in}\;\text{supernatant}) \end{array}}{\left(\text{Weight}\;\text{of}\;\text{enrofloxacin}\;\text{added}\right)}\times 100$$

$$\text{LC}\left(\%\right)=\frac{\left(\text{Weight}\;\text{of}\;\text{enrofloxacin}\;\text{in}\;\text{SLNs}\right)}{\left(\text{Weight}\;\text{of}\;\text{SLNs}\right)}\times 100$$

### 2.6. Evaluation of the DAS

#### 2.6.1. Determination of particle diameter, polydispersity index (PDI), and zeta potential

The diameter, PDI and zeta potential of the DAS was measured by photon correlation spectroscopy by using ZetasizerZX3600 (Malvern Instruments, UK) at 25 °C. The nanosuspension was diluted by 25 folds in distilled water for the determination of diameter and PDI, and 225 folds for the zeta potential to get the optimum kilo counts per second of 20–400 for the measurements (Xie et al., 2017). All measurements were repeated three times using samples from independent batches.

#### 2.6.2. Sedimentation rate

Briefly, the original height (H_0_) of 50 mL nanosuspension in 100 mL graduated cylinder after shaking was measured. The height (H) of the sediment in the cylinder after standing for 3 h was recorded. The sedimentation rate was calculated according to the equation: F = H/H_0_.

#### 2.6.3. Redipersibility

Fifty microliter nanosuspension was kept in a 100 mL graduated cylinder for one week, and then the layered nanosuspension was shocked under a magnetic shaker with rotation at 20 r/min to measure the re-dispersed time (Li et al., 2016).

#### 2.6.4. pH value

The pH value was determined by pH meter (DELTA 320, Mettler Toledo Co., Ltd, Shanghai, China) referring to the instruction.

#### 2.6.5. Stability studies

The stability of the DAS was measured using the accelerated stability test. The nanosuspension was stored at 40 ± 2 °C and 75 ± 5% relative humidity away from light for 1, 3, and 6 months. The nanosuspension was withdrawn periodically to measure its sedimentation rate, re-dispersibility, size, PDI, zeta potential, pH value, LC, and relatively labeled amount.

### 2.7. *In vitro* release studies

*In vitro* release profile of the nanosuspension was determined in PBS buffers (pH = 1.2 and 7.4) by using Dissolution tester RC806 (Tianjing Tiandatianfa Co., Ltd. China). The 0.1 mL nanosuspension (4 mg enrofloxacin) was added into in a dialysis bag and then placed in a dissolution cup containing 200 mL buffer at 37 °C under rotating propeller stirring at 100 rpm. One microliter samples were periodically taken from the receiver solution to measure the released drug and the same volume of fresh PBS was added after each sampling to keep a constant volume. The sink conditions were maintained for release study. The saturated concentration of enrofloxacin in pH = 7.4 PBS buffer was 116.5 μg/ml and higher in pH = 1.2 PBS buffer, in our test. The drug concentration in the release media is 20 μg/ml if the enrofloxacin released completely.

### 2.8. Detection of the injection site irritation

The injection site irritation of the nanosuspension in swine was observed by the self-comparison method. The nanosuspension was injected on the left gluteus medius of two pigs at the highest recommendation dosage (2.5 mg/kg) and the same amount of saline for injection was injected on the right gluteus medius. After medication, the clinical sign was observed every six hours. Two days post-injection, the animals were necropsied to evaluate the gross internal abnormalities at the injection site. The injection site muscle was collected for histopathological examination using the hematoxylin-eosin staining method.

### 2.9. Palatability

The palatability referred to the report of Tiwari et al. (Tiwari et al., 2017) Nine pigs from the same litter were randomly divided into three groups: control, enrofloxacin powder and nanosuspension groups with three pigs for each group. All pigs before the experiment were provided *ad libitum* access to pure water for 3 days to determine the normal water intake of each pig. Each pig in every group during the experiment period were provided *ad libitum* access to pure water, water containing enrofloxacin soluble powder or nanosuspension (responding to enrofloxacin concentration of 25 mg/L) for 3 days, respectively. At 9 o'clock every morning, the excess water (without or with the enrofloxacin at the dose of 5 mg per kg body weight) was added into the separate drinking water systems of each pig and the remaining water was measured at 9 pm of the next day. The amount of water intake every day of each pig was equal to the volume added at the day before minus the remaining volume.

### 2.10. Pharmacokinetic study

Evenly sexed twenty-four clinically healthy pigs were selected by veterinary for the pharmacokinetic study. The pigs before experiment were randomly divided into four groups with even sex and 6 animals in each group. Each group was randomly put into two pens with sex separate housing, and each pen with three pigs occupied 8 m^2^ space. The four groups were given the intramuscular injection of SLN suspension (2.5 mg/kg), intramuscular injection of 5% Baytril (2.5 mg/kg), intragastric administration of SLN suspension (5 mg/kg), and intragastric administration of enrofloxacin soluble powder (5 mg/kg), respectively. Blood samples (2 mL) were taken from anterior vena cava into heparinized tubes at fixed time points post administration and the enrofloxacin concentration in the plasma were determined by HPLC. The data on enrofloxacin concentrations in plasma-time were analyzed based on one-compartment model using the Winnonlin (Version 5.2.1, Pharsight Corporation, Mountain View, CA, USA) computer software.

### 2.11. HPLC assay

The enrofloxacin in plasma was extracted by mixing 0.5 mL plasma with 1 mL acetonitrile under the condition of oscillation for 3 min to assure a complete mixing, followed by centrifugation at 8000 rpm (3K15, Sigma, Germany) for 10 min at 4 °C. The supernatant after centrifugation was evaporated to dryness under a nitrogen evaporator (N-EVAP112; Organomation Associates Inc., USA) at 30 °C. The residue was reconstituted using 0.5 mL mobile phase and injected into HPLC with a Waters 2587 UV detector set at a wavelength of 278 nm for determination. The chromatographic separation was achieved with an analytical ZORBAX SB-2 C_18_ column (250 × 4.6 mm, i.d. 5 μm; Agilent Technology, USA) at 30 °C. The mobile phase was acetonitrile and 0.1% formic acid solution with the proportion of 14/86 (v/v) and a flow rate of 1.0 mL/min.

### 2.12. Statistical methods

The data on loading capacity, encapsulation efficiency, size, and PDI, zeta potential, intake water, blood drug concentrations, and pharmacokinetic parameters were expressed as mean ± standard error and analyzed using the SPSS 11.0 program for windows (SPSS Co., USA). Significance was evaluated at *p*-value of .05 using the one-way ANOVA by GraphPad Prism.

## 3. Results

### 3.1. Properties of DAS

**Figure 1.**
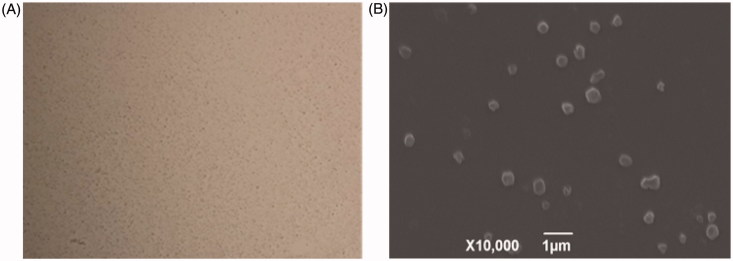
Photographs of enrofloxacin-loaded docosanoic acid SLN. A: Optical microscope (Magnification × 40); B: Scanning electron microscope (Magnification × 10000).

The nanosuspension was a milk-white uniform suspension with a sedimentation rate of 1 and pH value of 6, respectively. The re-dispersed time of layered suspension was only 55 s under a magnetic shaker rotating at 20 r/min. The optical microscope and scanning electron microscopy studies demonstrated that the SLNs were well dispersed with good particle size distributions and spherical (Figure 1). The mean EE, LC, sizes, PDI, and zeta potential of the optimal SLNs were 95.9%, 9.3%, 605 nm, 0.241, and −24.9 mv, respectively (Table 1).

**Table 1.** The characteristics of enrofloxacin-loaded docosanoic acid solid lipid nanoparticles (mean ± SD, *n* = 3).

MD(nm)	PDI	ZP (mv)	EE(%)	LC(%)
605.0 ± 4.9	0.241 ± 0.076	−24.9 ± 0.7	95.9 ± 1.6	9.3 ± 0.2

EE: Encapsulation efficiency; LC: Loading capacity; MD: Mean diameter; PDI: Polydispersity index; ZP: Zeta potentia.

### 3.2. Stability

After 6 months of storage at 40 ± 2 °C and 75 ± 5% relative humidity away from light, the LC, PDI and zeta potential of the nanoparticles were no evident change except a little increase in the size. The appearances, sedimentation rate and re-dispersibility of the suspension showed no significant changes except a slight decrease in drug contents in the six months as compared with the fresh preparation (Table 2).

**Table 2.** The stability of enrofloxacin-loaded docosanoic acid SLN suspension stored at 40 ± 2 °C and 75 ± 5% relative humidity away from light (mean ± S.D., *n* = 3).

Evaluation index	Initial preparation	1st month	2st month	3rd month	6th month
Appearances	White	White	White	White	White
Sedimentation rate	1 ± 0	1 ± 0	1 ± 0	1 ± 0	0.99 ± 0.01
Redispersibility (s)	55 ± 4	56 ± 6	55 ± 5	57 ± 5	57 ± 4
Size (nm)	605.0 ± 4.9	606.6 ± 4.7	610.5 ± 3.9	617.1 ± 5.3	619.5 ± 4.4^a^
PDI	0.24 ± 0.08	0.24 ± 0.02	0.26 ± 0.01	0.27 ± 0.03	0.29 ± 0.04
ZP (mV)	−24.9 ± 0.7	−25.3 ± 0.9	−24.6 ± 1.3	−24.5 ± 0.8	−25.2 ± 0.8
pH value	6	6	6	6	6
RLA (%)	99.77 ± 0.19	99.70 ± 0.18	99.62 ± 0.15	99.28 ± 0.18	99.02 ± 0.35^a^
LC (%)	9.3 ± 0.2	9.2 ± 0.4	9.4 ± 0.5	9.0 ± 0.3	9.0 ± 0.5^a^

PDI: Polydispersity index; ZP: Zeta potential; RLA: Relatively labeled amount; LC: loading capacity.

a Statistical significances compared with the initial preparation are *p* < .05.

### 3.3. *In vitro* release

**Figure 2.**
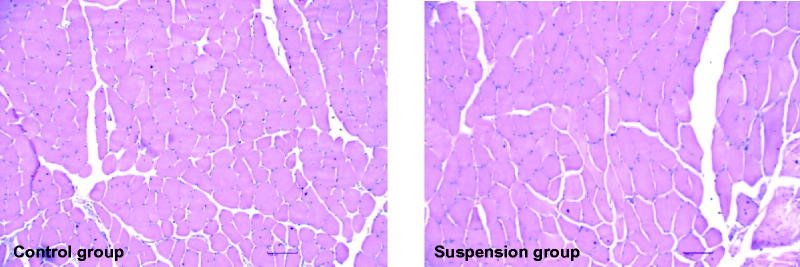
The pathological maps of gluteus medius of injection site. Left figure: Saline control group; Right figure: Enrofloxacin-loaded docosanoic acid SLN suspension.

*In vitro* release of enrofloxacin from the nanosuspension is illustrated in Figure 2. The DAS displayed a biphasic release pattern with a burst release of 25.76% within 4 h and a sustained release afterward in pH = 7.4 PBS. The nanoparticles released ∼97.82% at 72 h. In the native control, enrofloxacin was released about 60.51% within 4 h and completely at 24 h. The DAS also displayed a biphasic release pattern in pH = 1.2 PBS, but the rate of release is significantly swifter than those in pH = 7.4 PBS due to the larger solubility of enrofloxacin in acid solution.

### 3.4. Injection site irritation

**Figure 3.**
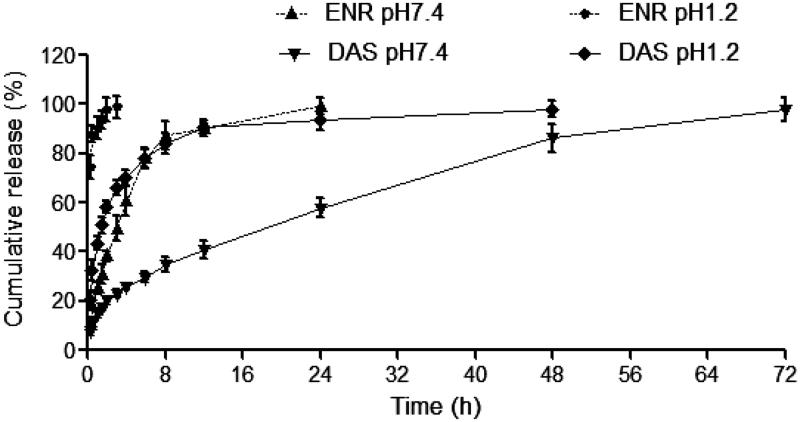
The release profiles of native enrofloxacin and enrofloxacin-loaded docosanoic acid SLN suspension in pH 1.2 and 7.4 PBS buffers. ENR: Native Enrofloxacin; DAS: Enrofloxacin-loaded docosanoic acid SLN suspension.

After intramuscular injection of the nanosuspension, the pigs did not show any abnormal clinical sign of pain or discomfort. No signs of inflammation were observed throughout the study. No macroscopic lesion and pathological changes at the injection site were detected through the necropsy and pathological studies (Figure 3).

### 3.5. Palatability

The statistical results of the normal water intake of pigs before and after administration are shown in Table 3. The water intake of pigs in the three groups did not differ before drug medication. The average daily normal water intake of each pig was in the range of 2.32–2.70 L/pig. The daily water intake of every pig in the nanosuspension group was about 2.74–3.06 L/pig, not significantly different with the control group and significantly higher than that of enrofloxacin powder group (1.31–1.62 L/pig) during the experiment period. The results suggested that the DAS mask the bitterness of enrofloxacin and improved the palatability. However, the daily water intake of pigs in DAS groups was sharply decreased to one-third of the normal level (0.84 ± 0.17 L) when the added concentration of enrofloxacin was increased threefold compared with the clinical maximum recommended dose.

**Table 3.** The daily water intake of different treatment groups.

Treatment	Before experiment			During experiment		
	1 day	2 day	3 day	1 day	2 day	3 day
Control	2.67 ± 0.33	2.56 ± 0.39	2.70 ± 0.46	2.67 ± 0.37	2.86 ± 0.48	2.82 ± 0.34
Enrofloxacin Powder	2.32 ± 0.35	2.54 ± 0.47	2.68 ± 0.52	1.62 ± 0.23^a^	1.45 ± 0.34^a^	1.31 ± 0.28^a^
Nanosuspension	2.56 ± 0.39	2.67 ± 0.33	2.67 ± 0.51	2.74 ± 0.35^b^	2.80 ± 0.43^b^	3.06 ± 0.27^b^

a Statistical significances compared with control are *p* < .05.

b Statistical significances compared with enrofloxacin powder are *p* < .05.

### 3.6. Pharmacokinetics

**Figure 4.**
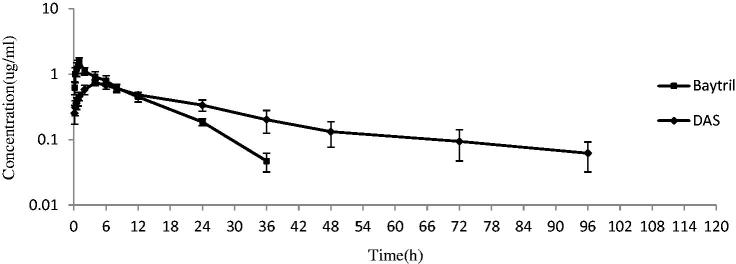
Mean plasma concentration-time curves of enrofloxacin after intramuscular injection at a dosage of 2.5 mg/kg. Baytril: Baytril solution; DAS: Enrofloxacin-loaded docosanoic acid SLN suspension.

**Figure 5.**
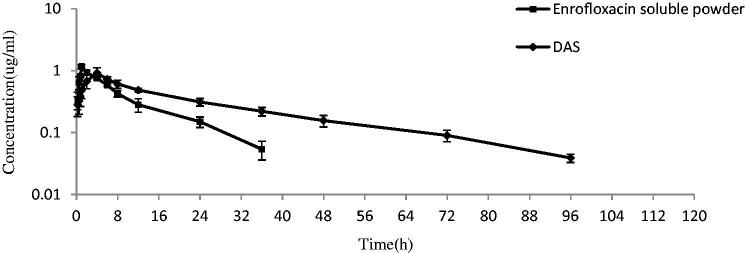
Mean plasma concentration-time curves of enrofloxacin after intragastric administration at a dosage of 5 mg/kg. DAS: enrofloxacin-loaded docosanoic acid SLN suspension.

The plasma enrofloxacin concentration was a good linear over the range of 0.05–2 μg/mL and the correlation coefficient was 0.9997. The LOQ was 0.04 μg/mL. The inter-day RSD for the three different concentrations (0.04, 0.08, and 2 μg/mL) were 6.3%, 5.6%, and 4.3%, respectively. The recovery from plasma for the three different concentrations was 91.9%, 96.0%, and 98.9%, respectively. Plasma enrofloxacin concentration-time curves after intramuscular and intragastric administration are shown in Figures 4 and 5, respectively. After intramuscular administration, the plasma drug in the nanosuspension group slowly reached a peak concentration of 0.75 ± 0.07 μg/mL at 4 h, then declined gently and sustained above 0.062 ± 0.03 μg/mL at 96 h (Figure 4). In contrast, the plasma enrofloxacin concentration in Baytril group swiftly achieved a higher peak value of 1.57 ± 0.22 μg/mL at 1 h, then decreased quickly and sustained above0.047 ± 0.01 μg/mL for 36 h (Figure 4). Compared with the Baytril injection, the elimination half-life (*T*_1/2ke_) and mean residence time (MRT) of enrofloxacin were enhanced by the docosanoic acid SLN from 6.33 to 20.05 h and from 11.27 to 37.76 h, respectively (Table 4). The AUC value of the nanosuspension was 1.6-fold higher than those of the Baytril injection (Table 4).

**Table 4.** The pharmacokinetic parameters of enrofloxacin-loaded docosanoic acid SLN (DAS) suspensions and baytril after intramuscular injection in swine at a dosage of 2.5 mg/kg.B.W.

Parameters	Unit	Baytril	Nanosuspension
AUC	h·μg/mL	13.60 ± 2.11	22.26 ± 6.26^b^
T½ka	h	0.17 ± 0.04	0.61 ± 0.23^b^
T½ke	h	6.33 ± 0.89	20.05 ± 5.20^b^
T_max_	h	0.90 ± 0.16	3.08 ± 0.73^b^
C_max_	μg/mL	1.36 ± 0.24	0.69 ± 0.06^b^
CL-F	L/h/kg	0.19 ± 0.03	0.12 ± 0.03^b^
MRT	h	11.27 ± 0.76	37.76 ± 4.46^b^
F (%)	**/**	**/**	163.62%

AUC: The area under the concentration-time curve; T½ka: The absorption half-life; T½ke: The elimination half-life; T_max_: The time to reach maximal drug concentration; C_max_: Maximal drug concentration; CL-F: Clearance rate; MRT: Mean residence time; F: The relative bioavailability.

a Statistical significances compared with Baytril solution are *p* < .05;

b Statistical significances compared with Baytril solution are *p* < .01.

After intragastric administration, the plasma drug concentration of enrofloxacin soluble powder increased rapidly to a peak value of 1.17 ± 0.08 μg/mL at 1 h, then swiftly dropped to 0.15 ± 0.03 μg/mL at 24 h and below the detection limit at 48 h (Figure 5). On the contrary, the plasma drug concentration of nanosuspension reached a slightly lower peak concentration of 0.92 ± 0.19 μg/mL at 4 h, and then fell slowly to 0.16 ± 0.03 μg/mL at 48 h and sustained above the LOQ until 96 h post administration (Figure 5). The pharmacokinetic analysis revealed that the DAS enhanced the bioavailability of enrofloxacin by 2.38 fold and extended the T_1/2ke_ from 5.58 to12.06 h and MRT from 12.33 to 35.15 h (Table 5).

**Table 5.** The plasma pharmacokinetic parameters of enrofloxacin-loaded docosanoic acid SLN suspension (DAS) and soluble powder after intragastric administration in swine at a dosage of 5 mg/kg.B.W.

Parameters	Unit	Soluble powder	Nanosuspension
AUC	h·μg/mL	9.89 ± 2.00	23.56 ± 3.67^b^
T_1/2 Ka_	h	0.31 ± 0.05	1.58 ± 0.84^b^
T_1/2 Ke_	h	5.58 ± 1.38	12.06 ± 5.47^a^
T_max_	h	1.42 ± 0.12	3.63 ± 0.83^b^
C_max_	μg/mL	1.03 ± 0.06	0.82 ± 0.15^a^
CL-F	L/h/kg	0.52 ± 0.08	0.22 ± 0.04^b^
MRT	h	12.33 ± 1.56	35.15 ± 0.54^b^
F(%)	**/**	**/**	238.31%

AUC: The area under the concentration-time curve; T½ka: The absorption half-life; T½ke: The elimination half-life; T_max_: The time to reach maximal drug concentration; C_max_: Maximal drug concentration; V-F: Apparent volume of distribution; MRT: Mean residence time; F: The relative bioavailability.

a Statistical significances compared with Baytril solution are *p* < .05.

b Statistical significances compared with Baytril solution are *p* < .01.

## 4. Discussion

Due to the bitter taste and poor aqueous solubility, the design of enrofloxacin formulations is very difficult, resulting in that there are no commercially available oral formulations for pigs (Martinez et al., 2006). In addition, the sustained release formulation is necessarily developed to enhance animal compliance and usage convenience (Haritova et al., 2003). In our previous study, the fatty acid SLNs are effective nanoparticle carriers for controlled release and enhanced bioavailability of enrofloxacin in mice. In our previous study, the 605.0 nm nanoparticles with zeta potential of −24.9 ± 0.7 mV possessed the highest encapsulation efficiency, loading capacity, transmembrane transport and intracellular retention performance (Xie et al., 2017). Therefore, the docosanoic acid SLNs in this study was studied as a potential sustained release formulation. SLNs are usually produced as either suspensions or dry powders. The suspensions are preferred with regard to the ease of handling (no reconstitution necessary) and for cost reasons (e.g. cost of the centrifuge and vacuum freeze-drying) (Mehnert & Mader, 2001). Cost is one of the most important considerations in veterinary clinical application to ensure the application of a novel formulation. In addition, the fatty acid SLN suspension was reported to have good stability (Wang et al., 2012). Therefore, the docosanoic acid SLNs in this study were prepared into nanosuspension and explored as a potential intramuscular injection and mixed drink because the intramuscular injection and oral are the most common and convenient drug administration in the modern intensive pig farms.

Its properties were investigated after preparing into nanosuspension. The suspension had an excellent sedimentation rate and re-dispersibility due to the small size with no agglomeration. The higher zeta potential of nanoparticles and steric hindrance of the polymers on the surface of the nanoparticles might both contribute to the excellent sedimentation rate and re-dispersibility. The good stability was observed as the polydispersity index, zeta potential, appearances, sedimentation rate, and re-dispersibility of the suspension had no significant changes after being stored at 40 ± 2 °C and 75 ± 5% relative humidity away from light for 6 months.

The sustained release performance was subsequently evaluated *in vitro*. *In vitro* release displayed a similar biphasic drug release pattern and a slower release compared to the native enrofloxacin. The biphasic drug release pattern suggested that the suspension would be a promising controlled release formulation. The initial fast release would be helpful to ensure the achievement of therapeutic concentrations timely, which is important for the therapeutic efficacy of concentration-dependent enrofloxacin. The following slow release would maintain effective therapeutic concentration *in vivo*.

Explored as an oral sustained release formulation, the palatability of the suspension is very important and should be evaluated initially. This palatability evaluation referred to the report of Tiwari et al. In the study, the daily intake amount of water was used to evaluate palatability of caffeine citrate formulation in mice (Tiwari et al., 2017). According to the clinic, maximum dosage of 5 mg/kg B.W. and the average daily water intake (about 3 L) of 15 kg pig, the final concentration of enrofloxacin in drink water was prepared into 25 mg/L for the nanosuspension and enrofloxacin powder groups to test palatability. The results demonstrated that the water intake of pigs in the nanosuspension group maintained the norm water intake as those of control group and was significantly higher than those of enrofloxacin powder group. These results suggested that the nanosuspension could significantly increase the palatability of enrofloxacin. However, the daily water intake of pig in nanosuspension groups was sharply decreased to one-third of the normal level (0.84 ± 0.17 L) when the added concentration of enrofloxacin was increased three-fold compared with the clinical maximum recommended dose. These results suggested that the palatability of the nanosuspension can be further improved by using other preparation techniques, such as enhancing the encapsulation efficiency and adding some flavoring agents. This study preliminary evaluated the palatability and it should be further studied in field trial to obtain more data. After intragastric administration, the extended systemic circulation of enrofloxacin with improved bioavailability was observed by DAS. This might be mainly because of the increased residence time and absorption of the nanoparticles in the gastrointestinal tract due to the small size and large surface area. The higher bioavailability and MRT could also be due to the increased permeability and an enhanced lymphatic uptake (Xie et al., 2010). These results demonstrated that the nanosuspension might be a promising oral prolonged release formulation with good palatability.

Developed as an injection sustained release formulation, the irritation at the injection site should be first investigated. The nanosuspension showed no irritation at the injection site at the clinical maximum recommended injection dose of 2.5 mg/kg due to the good biocompatibility of docosanoic acid and its proper pH value. After intramuscular administration, the sustained-release performance is evident compared with the baytril^®^ injection solution. In clinic, the dosage regimen of baytril^®^ injection solution was 2.5 mg enrofloxacin/kg bw per day for continuous 3–5 days. Based on the pharmacokinetics, the DAS might be reduced to a single dose or two doses of interval 2 days. The optimal dosage regimens will be determined by pharmacokinetics-pharmacodynamics analysis in future work. The improved bioavailability and extended systemic circulation might be due to the gradual release of entrapped drug from the lipid matrix and the form of a sustained release depot at the injection sites (Xie et al., 2010).

These results suggest that the DAS could achieve good controlled release and reduce dosing frequency by intramuscular administration and mixed-drinking. Although the blood drug concentration of the DAS formulation in this study is not very high at the recommended dose of the conventional preparations, the satisfactory drug concentration to achieve satisfactory therapeutic effect and minimize the emergence of antibiotic resistance could be obtained by the optimal dosage regimens determined by pharmacokinetics-pharmacodynamics analysis.

## 5. Conclusions

The DAS had uniform particle distribution, excellent sedimentation rate, re-dispersibility, good stability. The suspension had an excellent sustained-release with improved palatability and no irritation at the injection site. These results suggest that the suspension might be a promising formulation for oral and intramuscular administration in veterinary clinic.
